# Efficacy and safety of 125I seed implantation combined with thyroid-stimulating hormone suppression therapy in cervical lymph node metastases of differentiated thyroid carcinoma

**DOI:** 10.3389/fendo.2026.1747769

**Published:** 2026-05-08

**Authors:** Yi Ruan, Hui Yuan, Feng Zheng, Yang Yang, Xuehua Chen, Cheng Xu

**Affiliations:** 1 Department of Nuclear Medicine Changde Hospital Xiangya School of Medicine Central South University (The first people’s hospital of Changde city), Changde, Hunan, China; 2 Department of Radiological Physics Technology The Affiliated Cancer Hospital of Xiangya School of Medicine Central South University (Hunan Cancer Hospital), Changsha, Hunan, China

**Keywords:** 125I seed implantation, cervical lymph node metastasis, differentiated thyroid carcinoma, local control, survival analysis, thyroid-stimulating hormone suppression

## Abstract

**Background:**

Differentiated thyroid carcinoma (DTC) is a common endocrine malignancy, and cervical lymph node metastasis (LNM) represents the predominant pattern of disease recurrence. For patients who develop persistent or recurrent cervical lesions following total thyroidectomy and radioactive iodine (131I) therapy, conventional reoperation or additional 131I therapy often provides limited efficacy and may carry increased procedural risks.

**Methods:**

This single-center retrospective cohort study included 238 patients with persistent or recurrent cervical LNM after prior thyroidectomy and 131I therapy who were treated between January 2020 and January 2024. Among them, 118 patients received 125I seed implantation with thyroid-stimulating hormone (TSH) suppression. After 1:1 propensity score matching (PSM), 110 patients were included in each group for analysis. Short-term efficacy, thyroglobulin (Tg), radiologic changes, dosimetric consistency, survival outcomes, and adverse events were compared. Dynamic changes in Tg levels and the short-axis diameter of LNM were analyzed using a linear mixed-effects model.

**Results:**

After PSM, the partial response rates at 6 and 12 months were significantly higher in the 125I+TSH group. 125I+TSH therapy was an independent predictor of partial response. Patients in the 125I+TSH group exhibited a faster decline in Tg levels, with an average reduction of approximately 14.5% per month and a reduction in the short-axis diameter of LNM of 4.8% per month, corresponding to biochemical and morphological half-lives of 4.4 and 14.2 months, respectively. The overall incidence of adverse events was low.

**Conclusions:**

125I seed implantation combined with TSH suppression therapy accelerates Tg decline and lesion regression and demonstrates good safety.

## 1 Introduction

Differentiated thyroid carcinoma (DTC) is the most common malignant tumor of the endocrine system, accounting for more than 90% of all thyroid cancers (1). With advancements in imaging and pathological diagnostic techniques, its incidence has been steadily increasing. Although DTC generally carries a favorable prognosis (2), the postoperative cervical lymph node recurrence rate has been reported to range from 2% to 15% under current therapeutic and follow-up conditions, and may reach approximately 20%–30% in patients with extended follow-up durations or high-risk pathological features. Cervical lymph node metastasis (LNM) is therefore a major contributor to local recurrence and persistent disease (3). Current standard treatment for DTC consists of total thyroidectomy, radioactive iodine (131I) ablation (4), and long-term thyroid-sti mulating hormone (TSH) suppression therapy (5). Despite these treatments, a proportion of patients continue to exhibit residual or recurrent metastatic lesions even after receiving adequate doses of 131I therapy (6). These cases are referred to as radioiodine-refractory differentiated thyroid carcinoma (RAIR-DTC) (7), which presents a substantial clinical challenge due to the limited efficacy and elevated complication risks associated with repeat surgical intervention.

To improve local disease control in such patients, several local treatment modalities have been explored in recent years, including external beam radiotherapy, radiofrequency ablation, and percutaneous ethanol injection. However, these approaches often show limited long-term efficacy and may cause substantial damage to surrounding normal tissues (8–10). Permanent interstitial brachytherapy using 125I seeds is a low-dose-rate internal radiotherapy technique that enables precise irradiation of the target area while allowing rapid dose attenuation in adjacent normal tissues. This approach has shown promising therapeutic outcomes in several head and neck malignancies (11, 12). Continuous low-energy γ-ray emission from 125I seeds induces DNA strand breaks, apoptosis, and mitotic arrest in tumor cells, conferring advantages such as high dose concentration, minimal normal tissue injury, and excellent local control rates.

125I seed implantation is a feasible and safe option for the treatment of recurrent thyroid carcinoma lesions (12). However, most available studies are limited by small sample sizes, short follow-up periods, lack of control groups, and insufficient longitudinal evaluation (13). Moreover, combining 125I seed implantation with TSH suppression therapy may further enhance tumor control and survival outcomes through synergistic mechanisms involving radiotherapeutic effects and endocrine modulation. However, robust clinical evidence supporting this combined approach remains scarce.

Therefore, the present study aims to comprehensively evaluate the clinical efficacy, safety, and dosimetric consistency of 125I seed implantation combined with TSH suppression therapy in patients with postoperative cervical LNM of DTC. To improve the reliability of our findings, propensity score matching (PSM) and multi-model validation were employed. Our results may provide evidence supporting a precise, minimally invasive local therapeutic strategy for patients who are refractory to 131I therapy or unsuitable for repeat surgery.

## 2 Methods

### 2.1 Study design and ethics

This single-center, retrospective cohort study was conducted in accordance with the principles of the Declaration of Helsinki. The study protocol was approved by the institutional ethics committee (approval number: 2025-079-02). As this research involved the analysis of previously collected clinical data, the requirement for written informed consent was waived by the ethics committee. All patient data were anonymized prior to analysis to ensure strict protection of patient confidentiality.

### 2.2 Study population and enrollment process

**Figure 1:**
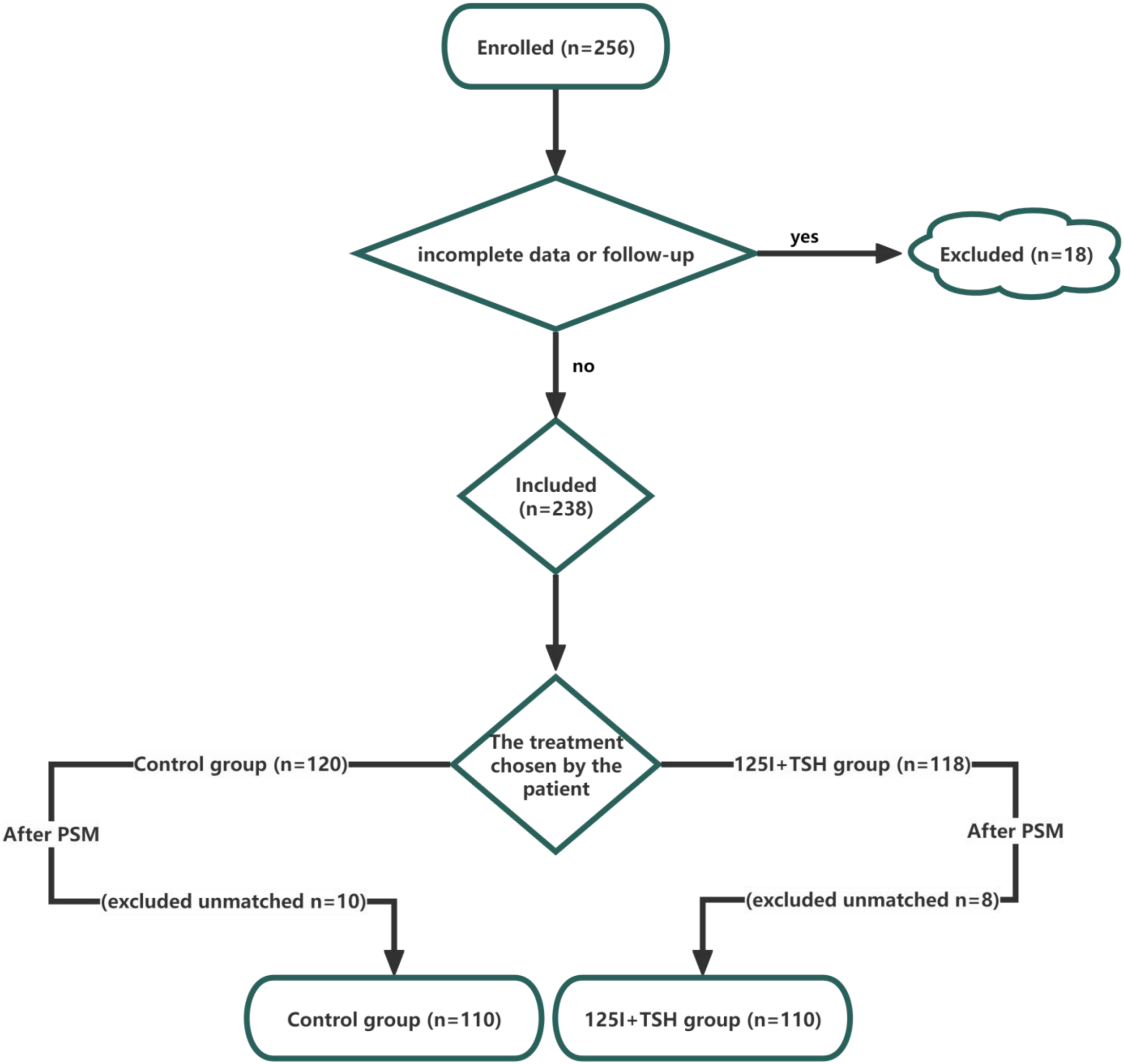
Flowchart of patient enrollment and grouping. A total of 256 patients were initially screened. Eighteen cases were excluded due to incomplete data or loss to follow-up, leaving 238 patients eligible for inclusion. Based on the treatment method selected by the patients, they were divided into the control group (n = 120) and the 125I+TSH suppression group (n = 118). After 1:1 PSM, 10 and 8 unmatched cases were excluded from each group, respectively, resulting in 110 patients in the control group and 110 patients in the 125I+TSH suppression group for the final analysis.

Patients with persistent or recurrent cervical LNM of DTC following prior thyroidectomy and 131I therapy who were treated at our institution between January 2020 and January 2024 were consecutively enrolled. As shown in **Figure 1**, a total of 256 cases were initially screened. Eighteen patients were excluded due to incomplete clinical records or loss to follow-up, leaving 238 eligible patients for analysis. Based on the treatment received, patients were divided into two groups: the control group (those who received 131I therapy or repeat surgery during the same period, n=120) and the 125I seed implantation combined with TSH suppression group (125I+TSH group, n=118). To minimize selection bias, 1:1 nearest-neighbor PSM was performed, yielding 110 matched cases in each group for the primary comparative analysis. RAIR-DTC was defined according to commonly accepted clinical criteria adapted from international guidelines, including: (1) absence of 131I uptake in metastatic lesions on post-therapy or diagnostic scans; (2) structural disease progression despite radioiodine uptake; or (3) persistent or recurrent disease after multiple courses of radioactive iodine therapy.

Inclusion criteria: (1) Postoperative DTC confirmed pathologically as papillary thyroid carcinoma (PTC) or follicular thyroid carcinoma (FTC); (2) Definitive diagnosis of cervical LNM confirmed by fine-needle aspiration (FNA), cytology, or pathology; (3) Presence of recurrent or persistent cervical LNM (radiologic or pathologic evidence) after standardized 131I therapy; (4) Clinical indication or patient preference: refusal of reoperation or inadequate response to previous 131I therapy requiring alternative local control measures; (5) Availability of complete follow-up data, including baseline and at least one follow-up evaluation for both biochemical and imaging parameters (from at least two of the following time points: 0, 1, 3, 6, and 12 months); (6) Agreement to receive TSH suppression therapy (dose individually adjusted by nuclear medicine physicians according to clinical guidelines) and signed informed consent for treatment.

At our institution, the decision to perform 125I seed implantation was made through multidisciplinary discussion involving nuclear medicine physicians, endocrinologists, and head and neck surgeons. The procedure was generally considered for patients with persistent or recurrent cervical LNM who were unsuitable for repeat surgery due to anatomical complexity or surgical risk, showed inadequate response to prior radioactive iodine therapy, or declined further surgical intervention. The number, size, and anatomical location of metastatic lymph nodes were evaluated to ensure suitability for image-guided local brachytherapy.

Exclusion criteria: (1) Pregnancy or lactation; (2) Active severe infection; (3) Coagulopathy defined as platelet count (PLT) < 50×10^9^/L, prothrombin time (PT) > 18 s, or prothrombin activity < 40%; (4) Severe dysfunction of major organs (heart, liver, or kidneys) based on clinical and laboratory evaluations; (5) Poor performance status: Karnofsky Performance Status (KPS) ≤ 60; (6) Presence of distant organ metastases (lung, bone, liver, brain, etc.) that could confound local efficacy assessment; (7) Irregular postoperative follow-up or missing key outcome data precluding valid efficacy/safety evaluation; (8) Any other conditions considered by investigators as unsuitable for 125I seed implantation or with poor follow-up compliance.

### 2.3 Treatment protocol and perioperative management

In 125I+TSH group, preoperative treatment planning was performed using a treatment planning system based on imaging to delineate the target volume and organs at risk. The prescribed dose and target D90 were determined, and the number, activity, and placement of seeds were calculated. Under ultrasound or computed tomography (CT) guidance, percutaneous implantation was performed using real-time imaging to achieve planned V100 coverage. Postoperatively, patients received TSH suppression therapy with individualized dose adjustments made by endocrinologists according to established guidelines. The 125I seeds were supplied by Saide Biological (Tianjin, China). Each seed had a diameter of 0.8 mm, a physical half-life of approximately 59.7 days, an effective tissue penetration diameter of about 1.7 cm, and an activity of 0.5-0.7 mCi. The prescribed target dose D90 ranged from 110–160 Gy. Implantation was performed under local anesthesia with an 18G needle under ultrasound guidance, avoiding major vessels and nerves. Post-procedure care included compression hemostasis and imaging verification of seed distribution.

In the control group, patients received standard clinical management and follow-up but did not undergo 125I seed implantation. Treatment strategies were determined according to institutional practice and multidisciplinary evaluation. Repeat surgery was considered for patients with surgically accessible lesions and acceptable operative risk, whereas additional radioactive iodine therapy was selected for patients with iodine-avid disease or biochemical recurrence. In selected cases with small or stable lesions, active surveillance was adopted. Specifically, 46 patients (38.3%) underwent repeat surgery and 74 patients (61.7%) received additional 131I therapy (**Supplementary Table S1**).

### 2.4 Follow-up and data collection

Patients were followed at baseline and 1 month, 3, 6, and 12 months after treatment. Laboratory assessments included serum Tg, thyroglobulin antibody (TgAb), TSH, free triiodothyronine (FT3), and free thyroxine (FT4). When clinically indicated, single-photon emission computed tomography/computed tomography (SPECT/CT) was also performed. Adverse events such as local pain, hematoma, recurrent laryngeal nerve palsy, hypocalcemia, thyroid function fluctuation, and infection were recorded.

### 2.5 Imaging and dosimetric evaluation

Within 24–72 hours postoperatively, cervical SPECT/CT (Siemens Symbia T16, Siemens Healthineers, Erlangen, Germany) was performed to verify seed distribution and dose coverage. Particular attention was paid to potential hotspots or cold zones and to consistency with the preoperative TPS plan.

Dosimetric parameters included (1) D90 (Gy): Minimum dose received by 90% of the target volume; (2) V100 (%): Percentage of the target volume receiving ≥ the prescribed dose; (3) Seed displacement (mm): Mean spatial deviation of seed centers between preoperative planning and postoperative imaging; and (4) Planned dose (PlannedDose proxy): Dose calculated by TPS or prescribed dose used as a surrogate variable in analysis.

Agreement between the planned dose and postoperative measured D90 was evaluated using the Bland–Altman method, reporting mean bias and 95% limits of agreement.

### 2.6 Outcome measures and definitions

Primary Outcome: (1) Short-term objective response (at 6 and 12 months), evaluated based on changes in the short-axis diameter of metastatic lymph nodes (according to RECIST version 1.1 lymph node criteria), combined with clinical and Tg dynamics. (2) Partial response (PR): ≥30% reduction in target lymph node short-axis diameter from baseline, without new lesions and with overall improvement. (3) Stable disease: Not meeting PR or progressive disease criteria. (4) Progressive disease: Appearance of new lesions or overall lesion enlargement. (5) The proportion of patients achieving PR was used as the primary efficacy endpoint.

Secondary Outcomes: (1) Survival outcomes: Local–regional recurrence-free survival (LRRFS) and PFS, calculated from the date of treatment; events were defined as local–regional recurrence/progression or any progression event, respectively. (2) Dynamic biochemical and imaging indicators: Longitudinal changes in log(Tg) and log(short-axis diameter). (3) Safety outcomes: Incidence and distribution of adverse events. (4) Dosimetric–implantation associations: Correlations between D90 and V100, planned dose, seed displacement, and multivariate regression analyses.

### 2.7 PSM

A logistic regression model was used to estimate propensity scores, with receipt of 125I+TSH therapy as the dependent variable. Covariates included age, sex, pathological subtype (PTC/FTC), lymph node region (central VI vs. lateral neck), baseline short-axis diameter, baseline Tg, TSH, FT3, FT4, TgAb positivity, cumulative 131I dose, and, where applicable, RAIR status. Matching was performed at a 1:1 ratio using nearest-neighbor matching without replacement and a caliper width of 0.2×standard deviation of the logit of the propensity score. Matching quality was assessed using Love plots and standardized mean difference (SMD), with SMD <0.1 considered indicative of adequate balance.

### 2.8 Statistical analysis

All statistical analyses were performed using SPSS version 26.0 (IBM Corporation, Armonk, NY, USA), R version 4.3.2 (R Foundation for Statistical Computing, Vienna, Austria), and GraphPad Prism 10 (GraphPad Software, San Diego, CA, USA). Continuous variables were tested for normality. Normally distributed variables were expressed as mean ± standard deviation (SD) and compared using the t-test, while non-normally distributed variables were expressed as median (interquartile range, IQR) and compared using the Mann–Whitney U test. Categorical variables were expressed as number (percentage) and compared using the χ² or Fisher’s exact test. Short-term efficacy was evaluated by comparing PR rates at 6 and 12 months using the χ² test. Independent predictors of PR were identified using multivariate logistic regression, and results were expressed as odds ratio (OR) with 95% confidence interval (CI). Model discrimination was assessed using receiver operating characteristic (ROC) curves. Dynamic changes in Tg and short-axis diameter were analyzed using a linear mixed-effects model (MixedLM), incorporating time, group, and their interaction terms. Time effects were further validated using two-way repeated-measures analysis of variance (RM-ANOVA). Survival outcomes (LRRFS and PFS) were analyzed using Kaplan–Meier curves with log-rank tests, and hazard ratios (HRs) with 95% CIs were reported. Dosimetric analyses were conducted using Pearson correlation and multivariate linear regression to examine associations between D90 and V100, seed displacement, and planned dose. Agreement between planned and measured doses was verified using the Bland–Altman method. All statistical tests were two-sided, with P < 0.05 considered statistically significant. To minimize bias, data were double-checked by two independent reviewers, and PSM was employed to control for confounding variables.

## 3 Results

### 3.1 Patient enrollment and PSM

A total of 256 eligible patients were initially screened. Eighteen patients were excluded due to incomplete records or loss to follow-up, leaving 238 eligible patients for analysis. Based on treatment strategy, 120 patients were assigned to the control group and 118 to the 125I+TSH group.

To minimize selection bias, 1:1 PSM was performed. Ten patients in the control group and eight in the 125I+TSH group were excluded because suitable matches were not identified. The final matched cohort included 220 patients (110 per group), which was used for primary comparative analyses of clinical outcomes, biochemical parameters, and imaging verification (**Figure 1**).

### 3.2 Baseline characteristics before and after PSM

**Figure 2:**
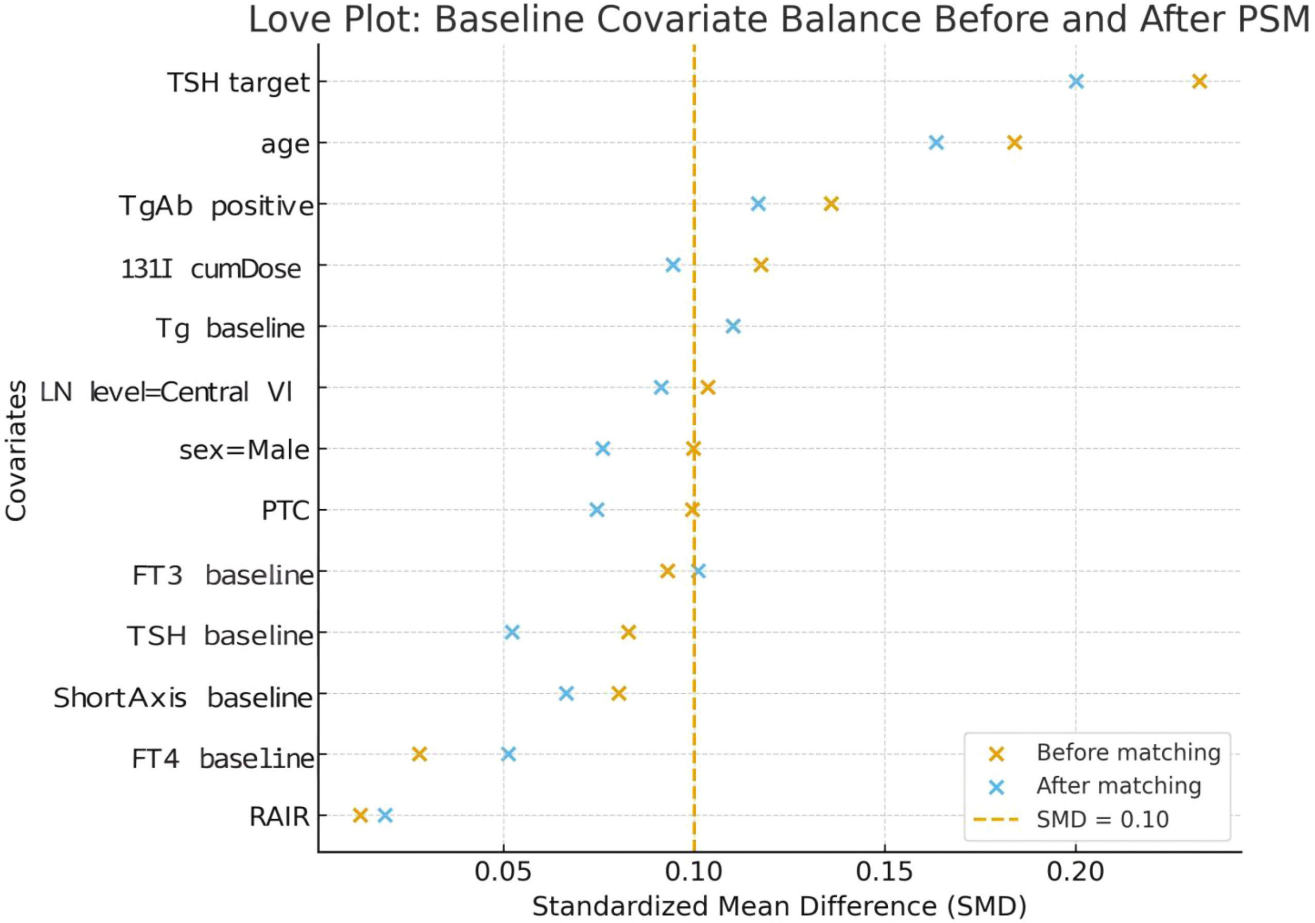
Love plots showing baseline covariate balance before and after PSM. After matching, the SMDs for all baseline variables were reduced to below 0.1, indicating good balance in covariate distribution between the 125I+TSH suppression group and the control group. Variables included TSH target value, age, TgAb positivity, cumulative 131I dose, baseline Tg, lymph node level, sex, pathology type, FT3, TSH, FT4, baseline short-axis diameter, and RAIR status.

Before PSM, moderate imbalance was observed in baseline characteristics such as TSH target levels, age, and cumulative 131I dose. After PSM, all baseline covariates achieved SMDs < 0.1, indicating successful matching and good comparability between groups (**Figure 2**).

Love plot visualization confirmed good balanced of thyroid function indices (TSH, FT3, FT4), Tg levels, and lymph node distributions after PSM, ensuring robust validity for subsequent analyses.

### 3.3 Comparison of baseline characteristics

Before PSM, 238 patients were included (control group: n = 120; 125I+TSH group: n = 118). There were no significant differences between groups in age, sex, pathology (PTC vs. FTC), or lymph node region (central vs. lateral neck) (all *p* > 0.05). Similarly, baseline biochemical indices (Tg, TgAb positivity, TSH, FT3, FT4) and cumulative 131I dose were also comparable between groups (**Table 1**). After PSM, 110 patients were included in each group. Baseline characteristics were well balanced, with no significant differences observed in age, sex, pathology, lymph node level, serum Tg, TgAb, TSH, FT3, FT4, or cumulative 131I dose between groups (all P > 0.05, SMD < 0.1), confirming successful matching (**Table 2**).

**Table 1:** Baseline characteristics of patients in the control and 125I+TSH groups before PSM.

Variable	Control group (n=120)	125I+TSH group (n=118)	*t/χ²/Z*	*P*
Age (years)	55.92 ± 11.72	53.81 ± 11.13	1.419	0.157
Sex, n (%)			0.593	0.441
Female	78 (65.0%)	71 (60.17%)		
Male	42 (35.0%)	47 (39.83%)		
Pathology, n (%)			0.588	0.443
PTC	111 (92.5%)	112 (94.92%)		
FTC	9 (7.5%)	6 (5.08%)		
Lymph node level, n (%)			0.638	0.424
Central area VI	64 (53.33%)	69 (58.47%)		
Lateral neck area	56 (46.67%)	49 (41.53%)		
ShortAxis baseline (mm)	12.66 ± 3.74	12.94 ± 3.27	-0.618	0.537
Tg baseline (ng/mL)	12.9 (7.33, 24.83)	13.4 (8.15, 26.43)	-0.477	0.633
TgAb positivity			1.099	0.294
Positive	20 (16.67%)	26 (22.03%)		
Negative	100 (83.33%)	92 (77.97%)		
TSH baseline (mU/L)	0.36 (0.09, 0.70)	0.435 (0.09, 0.75)	-0.884	0.377
FT3 baseline (pmol/L)	4.68 ± 0.64	4.74 ± 0.71	-0.718	0.474
FT4 baseline (pmol/L)	17.09 ± 3.00	17.17 ± 2.96	-0.216	0.829
Cumulative 131I dose (mCi)	138 (113.5, 181.5)	143.5 (122.75, 179.25)	-0.636	0.525

Data are presented as mean ± SD for normally distributed continuous variables, median (IQR) for non-normally distributed variables, and n (%) for categorical variables. Between-group comparisons were conducted using independent-samples *t* test, Mann–Whitney *U* test, or χ² test, as appropriate.

**Table 2:** Baseline characteristics of patients in the control and 125I+TSH groups after PSM.

Variable	Control group (n=110)	125I+TSH group (n=110)	*t/χ²/Z*	*P*
Age (years)	55.88 ± 11.48	53.95 ± 10.95	1.28	0.202
Sex, n (%)			1.553	0.213
Female	72 (65.45%)	63 (57.27%)		
Male	38 (34.55%)	47 (42.73%)		
Pathology, n (%)			0.736	0.391
PTC	102 (92.73%)	105 (95.45%)		
FTC	8 (7.27%)	5 (4.55%)		
Lymph node level, n (%)			0.463	0.496
Central area VI	60 (54.55%)	65 (59.09%)	0.463	0.496
Lateral neck area	50 (45.45%)	45 (40.91%)		
ShortAxis baseline (mm)	12.54 ± 3.75	12.95 ± 3.29	-0.866	0.388
Tg baseline (ng/mL)	12.85 (7.38, 23.85)	13.3 (8.15, 26.25)	-0.466	0.641
TgAb positivity			1.416	0.234
Positive	18 (16.36%)	25 (22.73%)		
Negative	92 (83.64%)	85 (77.27%)		
TSH baseline (mU/L)	0.36 (0.07, 0.69)	0.42 (0.08, 0.74)	-0.94	0.347
FT3 baseline (pmol/L)	4.68 ± 0.62	4.76 ± 0.71	-0.901	0.368
FT4 baseline (pmol/L)	17.03 ± 3.02	17.20 ± 2.89	-0.435	0.664
Cumulative 131I dose (mCi)	135 (112.25, 180)	143.5 (123, 179.25)	-1.032	0.302

Data are expressed as mean ± SD or median (IQR) for continuous variables, and n (%) for categorical variables. Between-group comparisons were performed using independent-samples *t* test, Mann–Whitney *U* test, or χ² test, as appropriate. All variables achieved satisfactory balance after PSM (SMD < 0.1).

### 3.4 Short-term treatment efficacy

Before PSM, the PR rate at 6 months was significantly higher in the 125I+TSH group than in the control group (68.6% vs. 20.0%, χ² = 57.103, *p* < 0.001). A similar difference was observed at 12 months (72.9% vs. 24.2%, χ² = 56.540, *p* < 0.001). The proportion of patients with stable disease was correspondingly lower in the 125I+TSH group at both time points (**Table 3**).

**Table 3:** Comparison of short-term efficacy between the control and 125I+TSH groups.

Time point	Response type	Control group (n=120)	125I+TSH group (n = 118)	χ²	*P* value
6 months	Partial response	24 (20.0%)	81 (68.64%)	57.103	<0.001
	Stable disease	96 (80.0%)	37 (31.36%)		
12 months	Partial response	29 (24.17%)	86 (72.88%)	56.540	<0.001
	Stable disease	91(75.83%)	32(27.12%)		

Data are expressed as n (%). PR, partial response; SD, stable disease. Comparisons between groups were performed using the χ² test.

After PSM, similar trends were observed. PR rates at 6 and 12 months remained significantly higher in the 125I+TSH group (70.0% vs. 19.1%, χ² = 57.705, *p* < 0.001; 73.6% vs. 23.6%, χ² = 55.041, *p* < 0.001). These findings indicate that 125I seed implantation combined with TSH suppression markedly enhances short-term tumor shrinkage and local disease control compared with conventional therapy (**Table 4**).

**Table 4:** Comparison of short-term efficacy between the control and 125I+TSH groups after PSM.

Time point	Response type	Control group (n=110)	125I+TSH group (n=110)	χ²	*P* value
6 months	Partial response	21 (19.09%)	77 (70.0%)	57.705	<0.001
	Stable disease	89 (80.91%)	33 (30.0%)		
12 months	Partial response	26 (23.64%)	81 (73.64%)	55.041	<0.001
	Stable disease	84 (76.36%)	29 (26.36%)		<0.001

Data are expressed as n (%). PR, partial response; SD, stable disease. Comparisons between groups were performed using the χ² test.

### 3.5 Multivariate logistic regression analysis

Multivariate logistic regression analysis was performed using the 6-month objective response rate (ORR_6m) as the outcome. Patients achieving complete response or PR were coded as 1, while those with stable or progressive disease (SD/PD) were coded as 0. Covariates included the same variables used in the PSM procedure. The results demonstrated that 125I combined with TSH suppression therapy was an independent predictor of objective response (**Table 5**).

**Table 5:** Multivariable logistic regression for predictors of treatment response (PR vs. SD).

Variable	β	SE	Wald	*p*-value	OR	95% CI lower limit	95% CI superior limit
125I+TSH (vs. Control)	2.253	0.319	49.888	0	9.514	5.092	17.777
Age (years)	0.009	0.014	0.441	0.506	1.009	0.982	1.037
Baseline short-axis (mm)	-0.059	0.047	1.572	0.21	0.942	0.859	1.034
Baseline Tg (ng/mL)	0.002	0.008	0.09	0.764	1.002	0.987	1.018
Baseline TSH (mU/L)	0.099	0.398	0.062	0.804	1.104	0.506	2.41
Baseline FT3 (pmol/L)	0.303	0.231	1.716	0.19	1.354	0.86	2.13
Baseline FT4 (pmol/L)	-0.06	0.053	1.265	0.261	0.942	0.849	1.045
Cumulative 131I dose (mCi)	-0.002	0.002	0.893	0.345	0.998	0.993	1.002
Sex (male=1)	-0.236	0.582	0.164	0.685	0.79	0.253	2.471
Pathology (PTC = 1)	-0.325	0.613	0.282	0.595	0.722	0.217	2.399
Lymph node level (central VI = 1)	-0.235	0.327	0.516	0.472	0.791	0.416	1.501
TgAb positivity (yes=1)	0.002	0.383	0	0.997	1.002	0.472	2.124
Intercept	-0.342	1.936	0.031	0.86	0.711	–	–

PR was coded as 1, SD as 0. “125I+TSH” indicates the treatment group (coded 1 vs. control 0). Continuous predictors are per 1-unit increase (short-axis: mm; Tg: ng/mL; TSH: mU/L; FT3/FT4: pmol/L; cumulative I-131 dose: mCi). OR with 95% CI are reported; Wald χ² used for significance.

In the overall cohort, 125I+TSH suppression therapy was significantly positively associated with 6-month ORR (β = 2.253, SE = 0.319, Wald = 49.888, *p* < 0.001), with an adjusted odds ratio (aOR) of 9.514 (95% CI: 5.092–17.777), indicating that patients receiving 125I+TSH therapy had approximately 9.5-fold higher odds of achieving an objective response compared to those not receiving this treatment. None of the other variables, including age, baseline lymph node size, baseline Tg, thyroid function indices, cumulative 131I dose, sex, pathological subtype, lymph node level, or TgAb positivity, were significantly associated with ORR_6m (all *p* > 0.05).

In the PSM-matched cohort (**Table 6**), the results were consistent (β = 2.370, SE = 0.341, Wald = 48.320, *p* < 0.001). 125I+TSH suppression remained an independent favorable factor for 6-month ORR, with an aOR of 10.701 (95% CI: 5.485–20.877). The comparable effect sizes observed in both cohorts indicate robust and stable results (all *p* > 0.05).

**Table 6:** Multivariable logistic regression analysis of predictors of PR after PSM.

Variable	β	SE	Wald	*p*-value	OR	95% CI lower limit	95% CI superior limit
125I+TSH (vs. Control)	2.37	0.341	48.32	0	10.701	5.485	20.877
Age (years)	0.011	0.015	0.559	0.455	1.011	0.982	1.042
Baseline short-axis (mm)	-0.061	0.05	1.478	0.224	0.941	0.853	1.038
Baseline Tg (ng/mL)	-0.003	0.008	0.127	0.722	0.997	0.981	1.013
Baseline TSH (mU/L)	-0.031	0.426	0.005	0.942	0.97	0.42	2.237
Baseline FT3 (pmol/L)	0.136	0.248	0.3	0.584	1.146	0.704	1.863
Baseline FT4 (pmol/L)	-0.077	0.057	1.86	0.173	0.926	0.829	1.034
Cumulative 131I dose (mCi)	-0.003	0.003	1.802	0.179	0.997	0.992	1.002
Sex (male=1)	-0.131	0.341	0.148	0.701	0.877	0.45	1.712
Pathology (PTC = 1)	0.809	0.669	1.464	0.226	2.246	0.606	8.328
Lymph node level (central VI = 1)	0.266	0.347	0.587	0.443	1.304	0.661	2.574
TgAb positivity (yes=1)	-0.027	0.409	0.004	0.948	0.974	0.437	2.168
Intercept	-0.227	2.162	0.011	0.916	0.797	–	–

PR was coded as 1 and SD as 0. “125I+TSH “ indicates the combined therapy group (coded 1 vs. control 0). Continuous variables were entered as per-unit increases. Adjusted odds ratios (ORs) with 95% confidence intervals (CIs) were calculated. Variables with *P* < 0.05 were considered statistically significant.

### 3.6 Predictive performance of the response model

**Figure 3:**
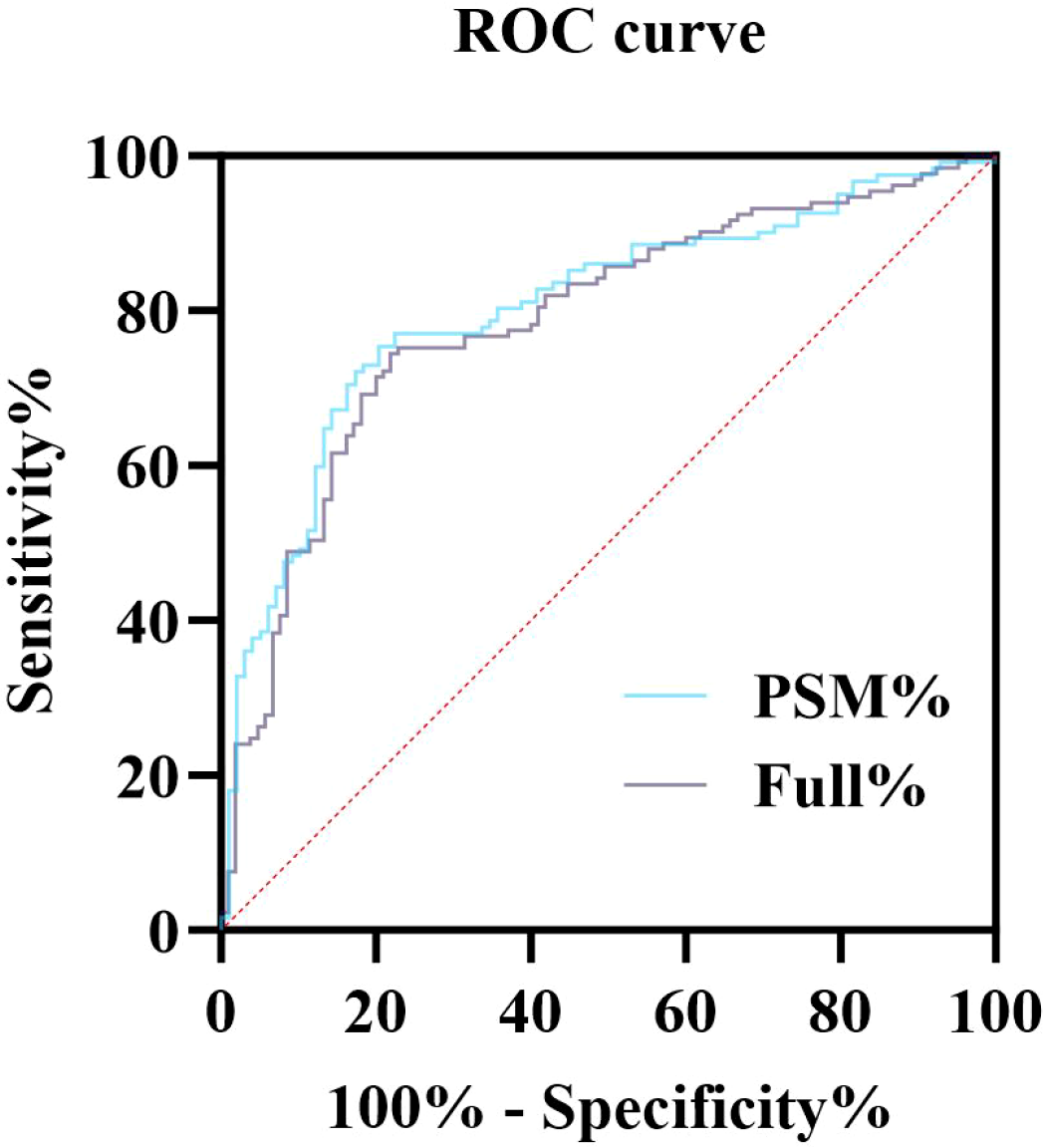
ROC curves for predicting PR before and after PSM. Both pre- and post-PSM models demonstrated good discriminatory ability, with AUC values of 0.786 and 0.803, respectively. After PSM, the model’s predictive accuracy was slightly improved, achieving an optimal balance between sensitivity (75.4%) and specificity (79.6%).

ROC curve analysis demonstrated good discriminatory ability for predicting treatment response both before and after PSM. The pre-PSM model yielded an AUC of 0.786 (95% CI: 0.728–0.845), with sensitivity 74.4% and specificity 78.1%. After PSM, the model performance slightly improved, with an AUC of 0.803 (95% CI: 0.744–0.862), with sensitivity 75.4% and specificity 79.6%. These results indicate that PSM effectively enhances model performance by reducing baseline bias (**Table 7**; **Figure 3**).

**Table 7:** ROC analysis of the predictive performance before and after PSM.

Variable	AUC	Youden	S.E.	95%CI	Sensitivity (%)	Specificity (%)
PSM cohort	0.8029	0.55	0.02997	0.7441 to 0.8616	75.41	79.59
Full cohort	0.7864	0.5254	0.02992	0.7277 to 0.8450	74.44	78.1

AUC: area under the receiver operating characteristic curve; SE: standard error; CI: confidence interval. Sensitivity and specificity were determined based on the optimal Youden index.

### 3.7 Mixed-effects model analysis of Tg and lymph node size

In the linear mixed-effects model evaluating longitudinal changes in log-transformed Tg levels, the control group showed a significant time-dependent decline (Time coefficient = −0.044/month, 95% CI −0.065 to −0.022, *p* < 0.001), corresponding to an approximate monthly decrease of 4.3%.

**Figure 4:**
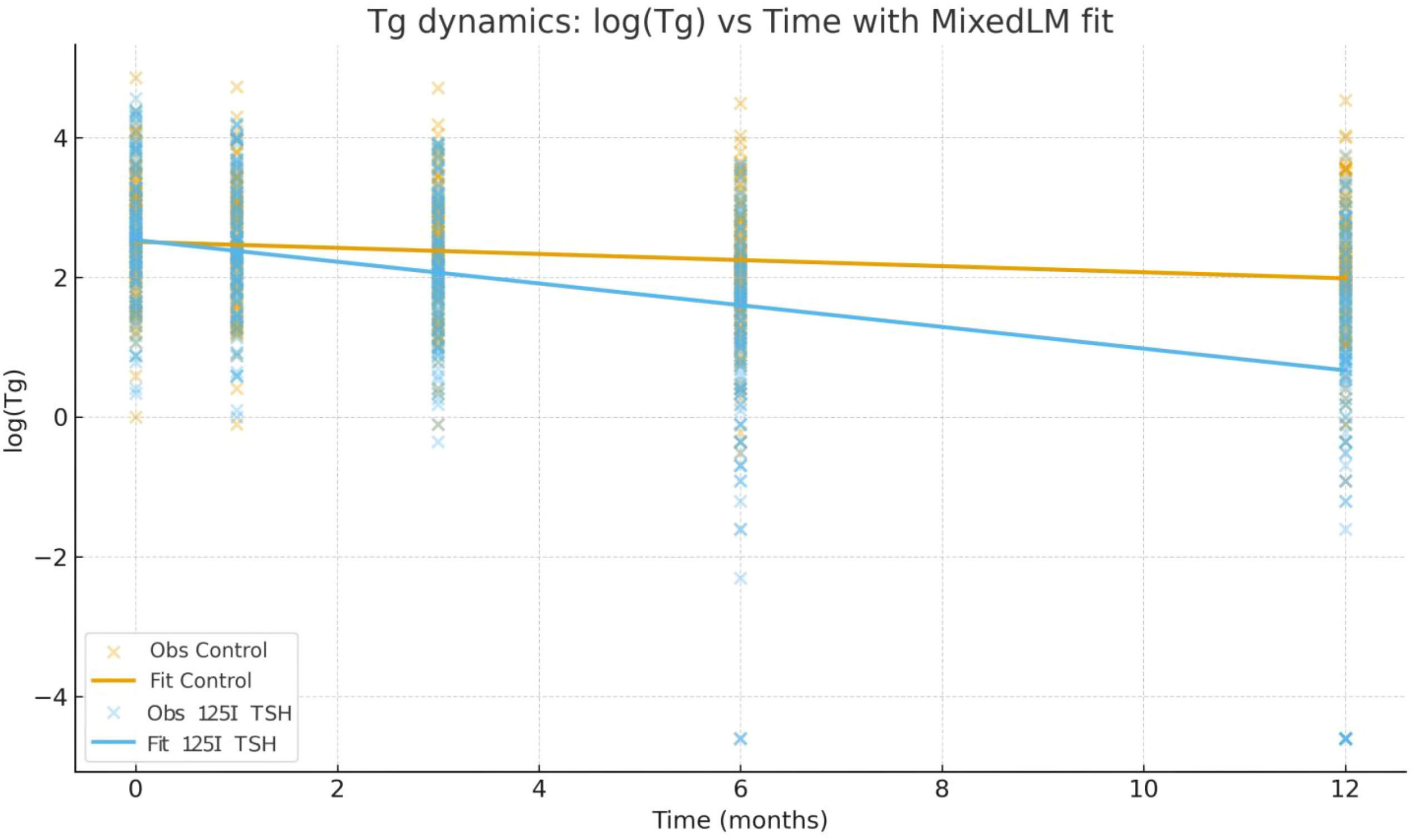
Tg dynamics by treatment group. Observed log(Tg) values at each time point and the fitted curves from the MixedLM are shown.

Compared with controls, the 125I+TSH group showed a lower baseline Tg level (β = −0.311, P = 0.011) and a significantly steeper decline over time (interaction β = −0.112, P < 0.001), resulting in an overall slope of −0.156 per month (~14.5% monthly reduction). Model projections indicated Tg reductions of approximately 23% and 41% at 6 and 12 months in the control group, compared with 61% and 85% reductions in the 125I+TSH group. The estimated biochemical half-life of Tg was 15.8 months in the control group and 4.4 months in the 125I+TSH group (**Table 8**; **Figure 4**).

**Table 8:** linear mixed-effects model for log(Tg) over time.

Term	β (estimated)	SE	95% CI (lower–upper)	P value
Intercept	2.377	0.086	2.209–2.544	<0.001
Time c(per month)	-0.044	0.011	−0.065 to −0.022	<0.001
125I+TSH (vs Control)	-0.311	0.122	−0.549 to −0.072	0.011
Time c × 125I+TSH	-0.112	0.016	−0.142 to −0.081	<0.001
Random effects	Variance/Covariance	SE	95% CI	P value
Group Var (random intercept)	6.109	0.672	4.791–7.426	<0.001
Time_c Var (random slope)	0.094	0.011	0.072–0.115	<0.001
Group × Time_c Cov	0.234	0.056	0.124–0.344	<0.001

The model is a LMM with the formula: log(Tg) ~ Time_c+Group+Time_c × Group+(Time_c | ID). Time_c represents the centered time variable; Group indicates treatment group (125I+TSH vs. Control); random effects include individual-specific intercepts and slopes. Negative coefficients for Time_c and the interaction term indicate a decline in Tg over time, with a more pronounced decrease in the 125I+TSH group. Group Var, Group×Time_c Cov, and Time_c Var represent the variance of the random intercept, the covariance between intercept and slope, and the variance of the random slope, respectively. Outcome is log(Tg); Time_c in months (0, 1, 6, 12); fixed effects include Group and its interaction with Time_c. Coefficients are on the log scale; negative values indicate a decline.

**Figure 5:**
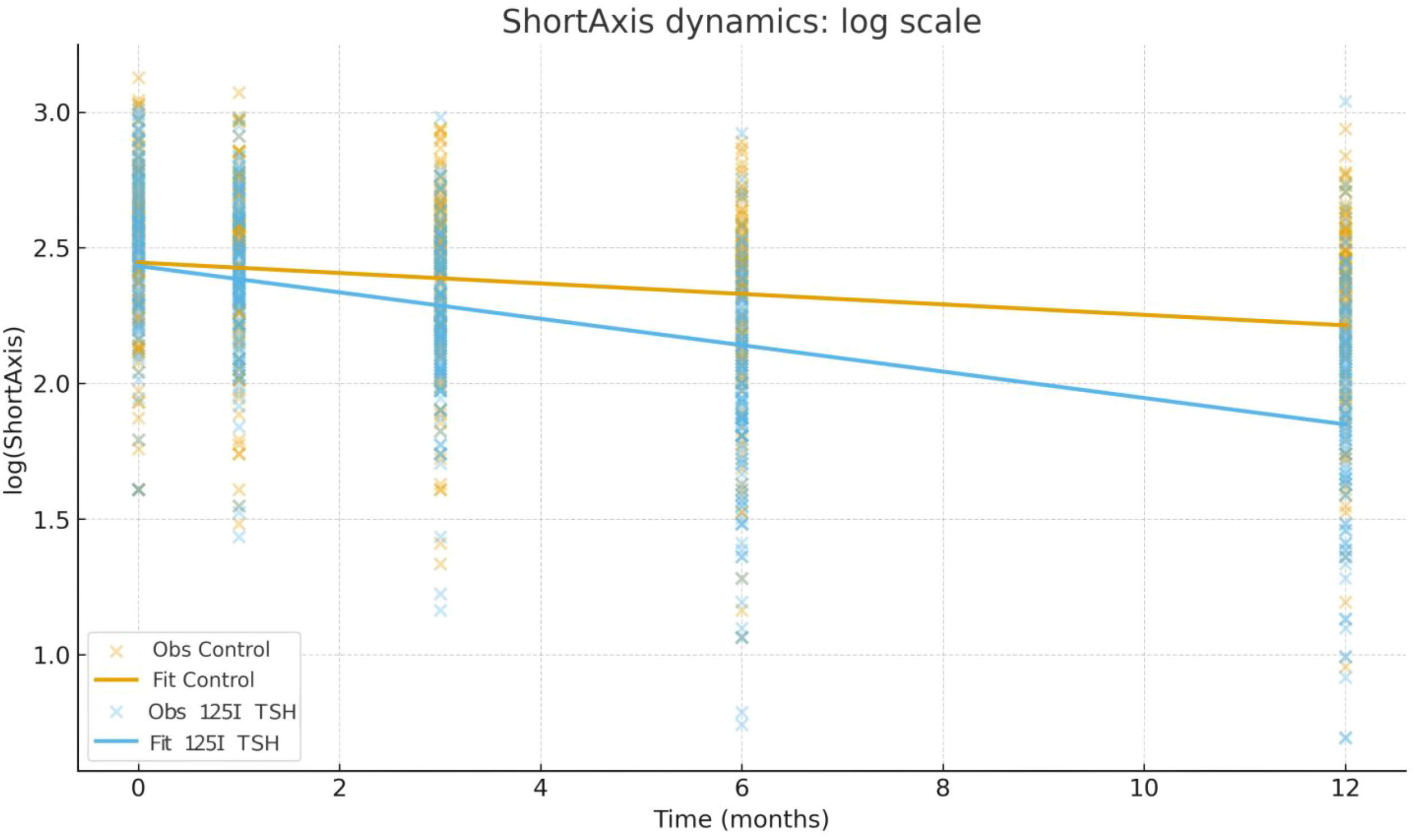
Short-axis dynamics on the log scale by treatment. Scatter points show observed log(ShortAxis) values; solid lines depict MixedLM fits. The 125I+TSH group exhibits a significantly steeper decline over time than controls (interaction *P* < 0.001).

For lymph node short-axis diameter, the control group also showed a modest time-dependent decrease (β = −0.0193 per month, P < 0.001). In contrast, the 125I+TSH group exhibited a greater baseline reduction (β = −0.1008, P = 0.011) and a significantly steeper decline (interaction β = −0.0294, P < 0.001), corresponding to an overall monthly reduction of approximately 4.8%. Estimated half-lives for lymph node shrinkage were 35.9 months in the control group and 14.2 months in the 125I+TSH group (**Table 9**; **Figure 5**).

**Table 9:** Linear mixed-effects model for log(ShortAxis) over time.

Term	β (estimated)	SE	95% CI (lower–upper)	P value
Intercept	2.3877	0.028	2.3328–2.4426	<0.001
Time c(per month)	-0.1008	0.0398	−0.1788 to −0.0228	0.0113
125I+TSH (vs Control)	-0.0193	0.0021	−0.0235 to −0.0151	<0.001
Time c × 125I+TSH	-0.0294	0.003	−0.0353 to −0.0234	<0.001
Random effects	Variance/Covariance	SE	95% CI	
Group Var (random intercept)	7.0813	0.7754	5.5615–8.6011	<0.001
Time_c Var (random slope)	0.0318	0.0045	0.0230–0.0407	<0.001
Group × Time_c Cov	0.1643	0.0384	0.0890–0.2395	<0.001

Outcome: log(ShortAxis); *Time_c* in months (0/1/6/12). Negative β indicates a decline.

### 3.8 Dynamic changes in Tg and lymph node size

**Figure 6:**
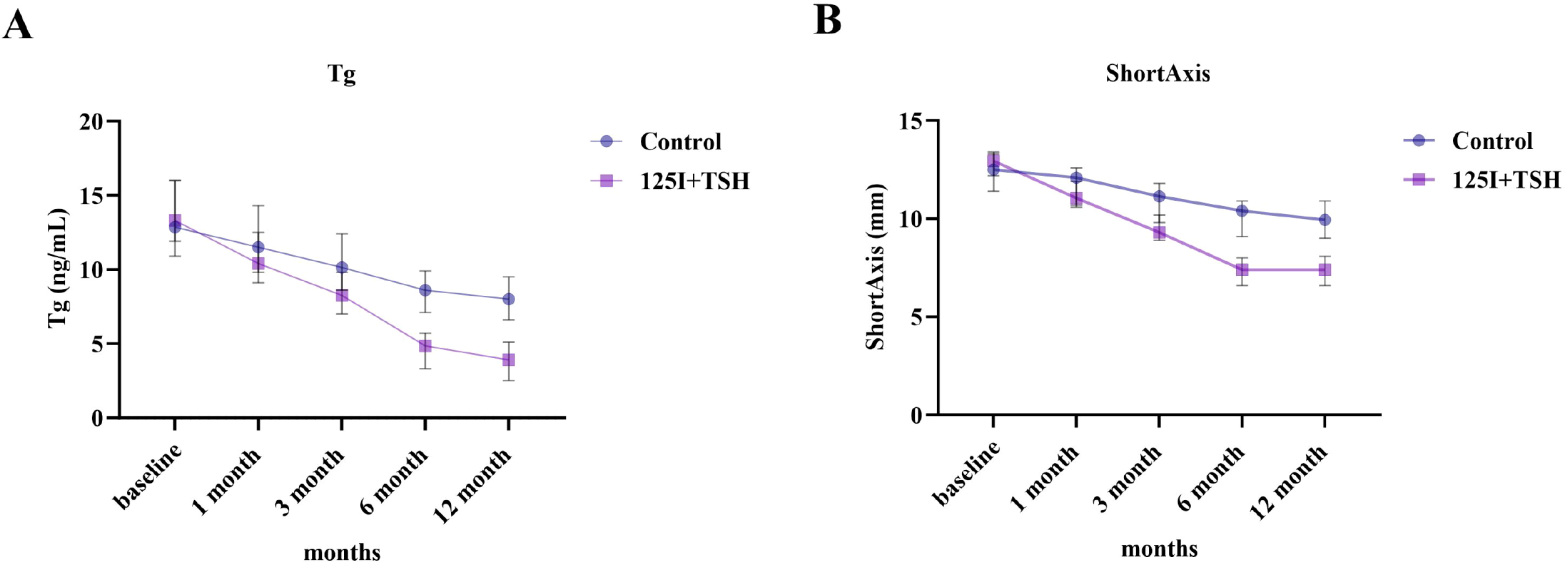
Temporal evolution of serum Tg and LN short-axis over 12 months. **(A)** Trends in serum Tg (ng/mL) from baseline to 12 months; **(B)** Trends in metastatic LN short-axis diameter (mm) over the same period. Data are presented as mean ± standard error (SE).

Continuous measurements of serum Tg and metastatic LN short-axis diameter showed progressive declines over 12 months in both groups, with markedly greater reductions in the 125I+TSH group (**Figure 6**). At 12 months, Tg levels in the 125I+TSH group decreased by approximately 80–85% from baseline, and mean short-axis diameter decreased by approximately 40–45%, compared with approximately 40–45% and 20–25% reductions, respectively, in the control group.

Two-way repeated-measures ANOVA demonstrated significant effects of time and group, with significant time × group interactions for both Tg and short-axis diameter (**Table 10**). For Tg, time accounted for the largest contribution (7.06%, *p* < 0.0001), with significant intergroup differences (*p* = 0.0051) and a statistically significant interaction (*p* = 0.0235), indicating a faster decline in the 125I+TSH group during follow-up. For short-axis diameter, time contributed 18.05% (*p* < 0.0001), with significant group (*p* = 0.0042) and interaction effects (*p* < 0.0001), suggesting more pronounced morphological shrinkage in the 125I+TSH group. These results are consistent with the mixed-effects model and longitudinal trend plots (**Figure 6**), further confirming the time-dependent advantage of 125I+TSH combination therapy.

**Table 10:** Two-way repeated-measures ANOVA for time, group, and their interaction (PSM).

Variation	Source of Variation	% of total variation	P value	P value summary
Tg	Time × Group	0.8698	0.0235	*
	Time	7.063	<0.0001	****
	Group	0.6031	0.0051	**
	Subject	49.81	0.0185	*
ShortAxis	Time × Group	2.402	<0.0001	****
	Time	18.05	<0.0001	****
	Group	2.677	0.0042	**
	Subject	69.67	<0.0001	****

*p < 0.05, **p < 0.01, ***p < 0.001, ****p < 0.0001.

### 3.9 Survival outcomes

**Figure 7:**
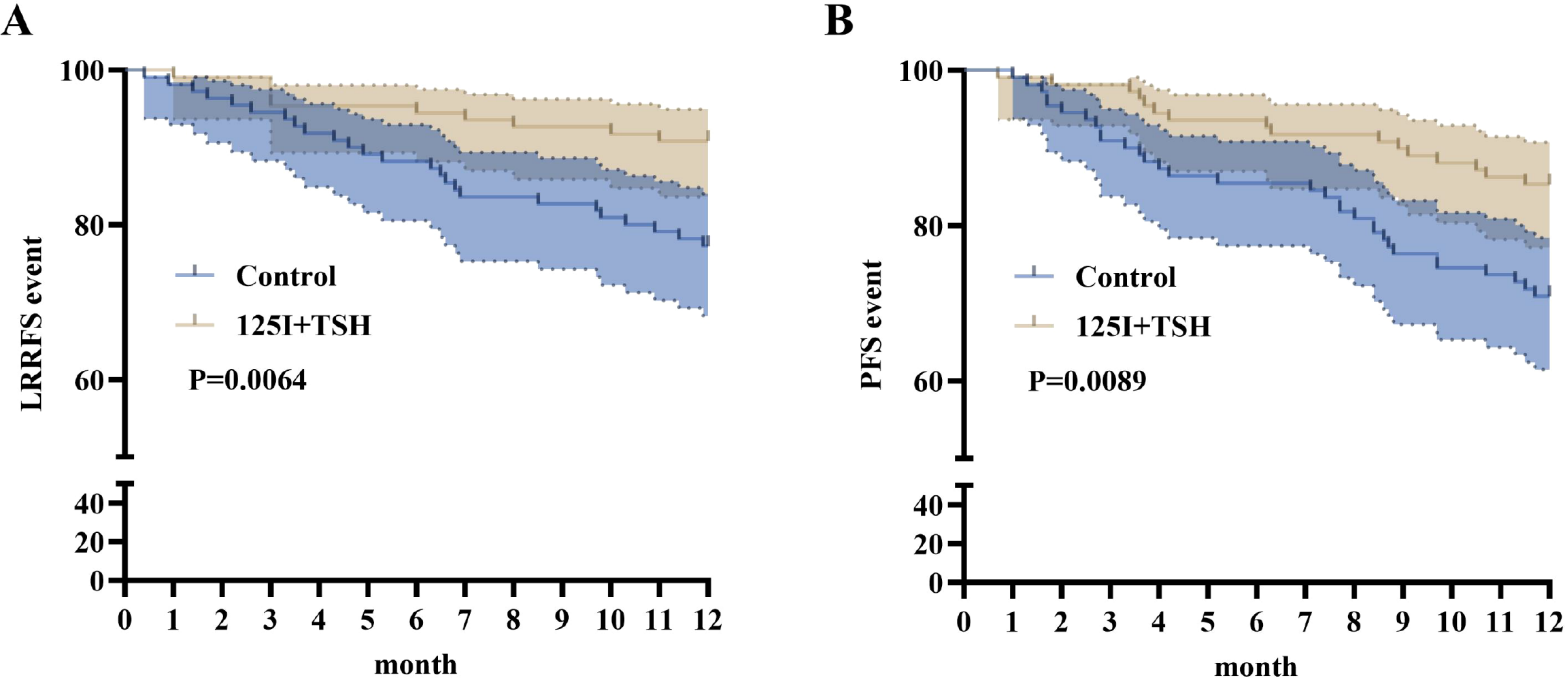
Kaplan–Meier survival curves. **(A)** LRRFS; **(B)** PFS. 125I+TSH group (light brown curve) and Control group (blue curve). Shaded areas represent 95% CIs.

Kaplan–Meier analysis showed 12-month LRRFS rates of 91.8% in the 125I+TSH group versus 79.5% in controls (*p* = 0.0064, HR = 2.662, 95% CI: 1.372–5.165), and PFS rates of 88.7% versus 74.9% (*p* = 0.0089, HR = 2.180, 95% CI: 1.238–3.841). These results indicate that 125I+TSH therapy significantly improves local–regional recurrence-free and progression-free survival compared with standard treatment (**Figure 7**; **Table 11**).

**Table 11:** Comparison of survival outcomes between groups (log-rank test).

Outcome	Hazard ratio (125I+TSH vs. Control)	95% CI	χ²	P value
LRRFS	2.662	1.372 – 5.165	7.427	0.0064
PFS	2.18	1.238 – 3.841	6.838	0.0089

LRRFS, locoregional recurrence-free survival; PFS, progression-free survival. Hazard ratios >1 indicate higher event-free probability in the 125I+TSH group using Kaplan–Meier analysis with log-rank test.

### 3.10 Imaging validation and dosimetric consistency

#### 3.10.1 Imaging validation

**Figure 8:**
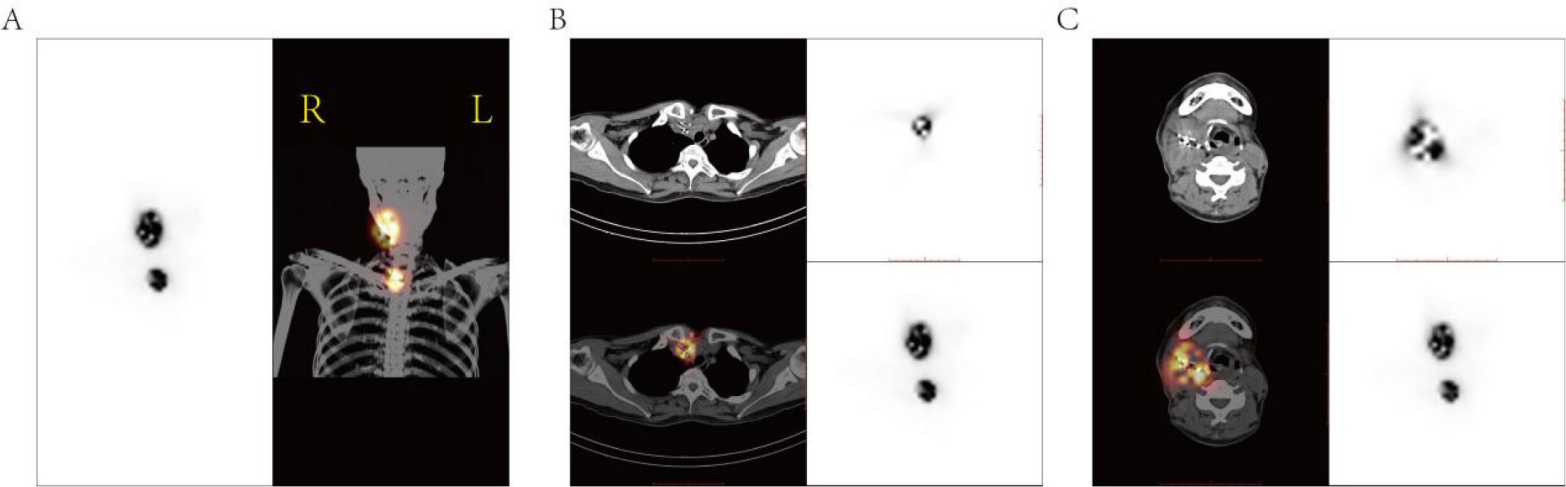
SPECT/CT fusion imaging of post-125I implantation radiation dose distribution. **(A)** Coronal SPECT/CT fused image from whole-body 125I WBS (anterior view); **(B)** transverse view; **(C)** axial view, showing radioactive uptake primarily localized to the thyroid bed and regional LNs.

Postoperative SPECT/CT showed that 125I seed distribution was highly consistent with preoperative TPS planning. Radioactivity was confined to the thyroid bed and cervical lymph node areas without abnormal uptake in distant organs. Dose distribution achieved full target coverage without evident hot or cold spots, confirming precise implantation and effective dose delivery (**Figure 8**).

#### 3.10.2 Relationship between D90, dosimetric parameters, and implantation accuracy

**Figure 9:**
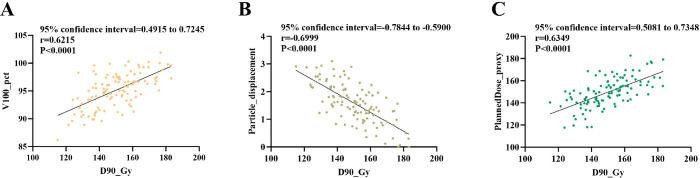
Correlation analysis of D90 with dose distribution and physical parameters. **(A)** Correlation between D90 and V100 (percentage of target volume receiving ≥100% of prescribed dose); **(B)** correlation between D90 and seed displacement (mm); **(C)** correlation between D90 and planned dose (Gy). Each point represents a patient; solid lines indicate linear regression fit, and shaded areas represent 95% confidence intervals. Pearson correlation coefficient (R) and P values are indicated.

**Figure 10:**
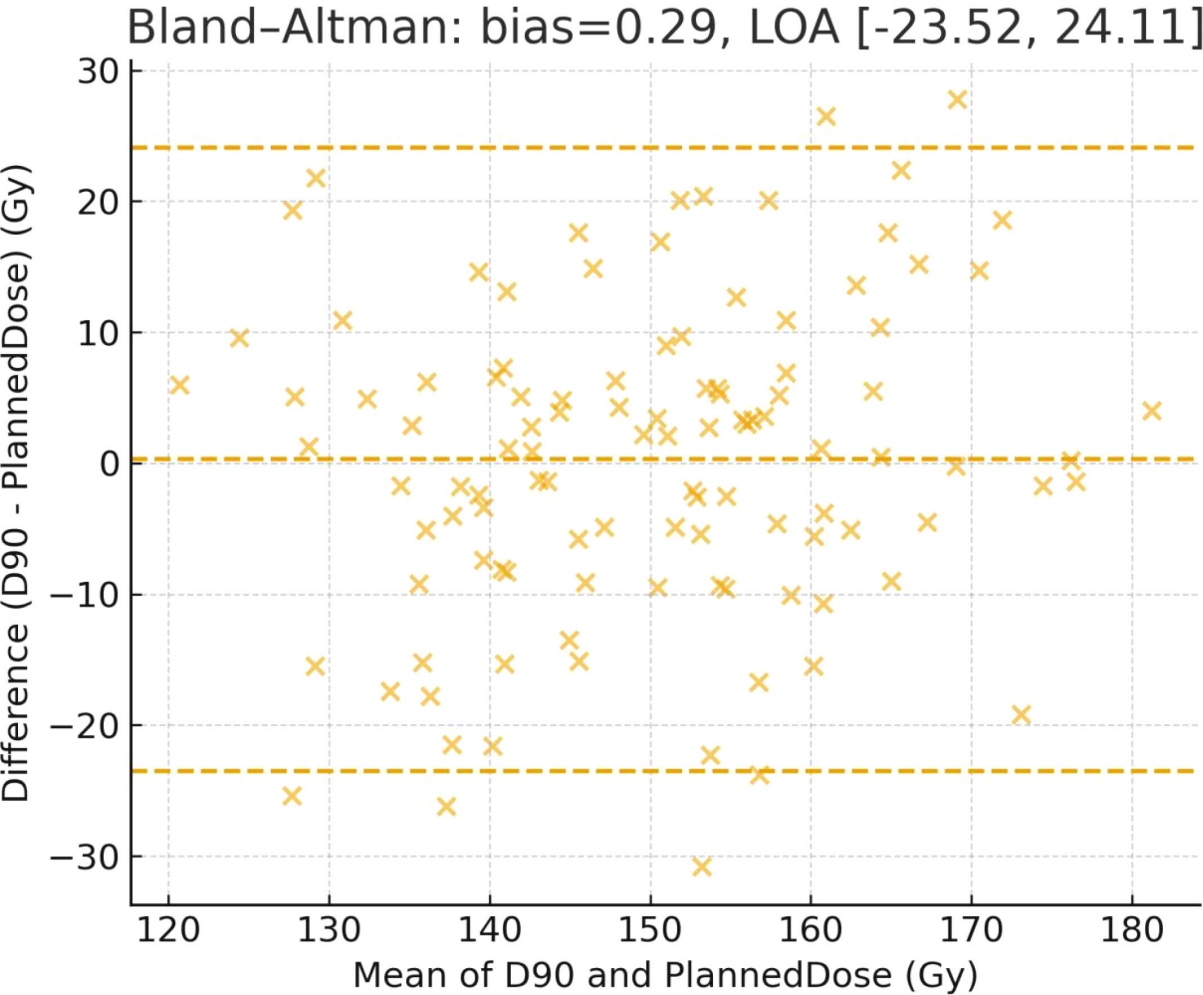
Bland–Altman plot comparing planned dose and measured D90. Dashed lines indicate the mean bias (center line) and the 95% limits of agreement (upper and lower lines).

Higher D90 correlated positively with V100 (r = 0.615, *p* < 0.001) and Planned Dose (r = 0.640, P < 0.001), but negatively with Seed Displacement (r = −0.6899, *p* < 0.001). Agreement between planned and measured D90 was confirmed using Bland-Altman analysis (mean bias: 0.29 Gy; 95% limits of agreement: −23.52–24.11 Gy). Multivariate linear regression identified V100 (β = 1.081, *p* = 0.001) and Planned Dose (β = 0.403, *p* < 0.001) as independent positive predictors of D90, while Seed Displacement was a strong negative factor (β = −9.983, *p* ≈ 10^-^¹²). The model fit was good (R² = 0.710, adjusted R² = 0.693), explaining ~71% of total variance. These findings highlight that higher planned doses and precise seed placement are key determinants of optimal dosimetric uniformity (**Tables 12**, **13**; **Figures 9**, **10**).

**Table 12:** Model fit and agreement statistics.

Metric	Value
R²	0.71
Adjusted R²	0.693
AIC	784.252
BIC	803.156
Bland–Altman bias	0.294
95% limits of agreement	−23.519 to 24.106

Goodness-of-fit and agreement between paired measurements (e.g., planned vs. measured D90). *R²/Adjusted R²* quantify explained variance; *AIC/BIC* assess parsimony (lower is better). Bland–Altman *bias* is the mean difference, *LOA = bias ± 1.96×SD* of differences.

**Table 13:** Multivariable linear regression for predictors of measured D90 (Gy).

Predictor	Coef. (β)	Std. Err.	*t*	*P*	95% CI
Intercept	2.935	28.186	0.1	0.917	−52.965 to 58.835
V100 (%)	1.081	0.319	3.39	0.001	0.449 to 1.713
Particles per node (n)	0.032	0.093	0.35	0.729	−0.153 to 0.217
Needle count (n)	0.195	0.4	0.49	0.627	−0.599 to 0.989
Particle displacement (mm)	−9.983	1.229	−8.13	1.0×10^-^¹²	−12.419 to −7.546
PlannedDose (proxy, Gy)	0.403	0.071	5.65	1.46×10^-7^	0.261 to 0.544
RPU count	~0	~0	−0.46	0.645	—

Dependent variable: measured D90 (Gy). Coefficients are per-unit change in the predictor (e.g., per 1% V100, per 1 mm displacement, per 1 Gy planned dose). Significant predictors are bolded.

### 3.11 Safety and adverse events

Both treatment groups demonstrated good tolerability, and no severe or life-threatening adverse events were observed (**Table 14**). The overall incidence of any-grade adverse events was 15.45% in the 125I+TSH group and 22.73% in controls (P = 0.170). Most events were mild and reversible. Local pain and minor hematoma were most common in both groups, whereas recurrent laryngeal nerve paresis and hypocalcemia occurred mainly in controls (8.18% and 5.45%, respectively) and were rare or absent in the 125I+TSH group (0.91% and 0.00%). No thyroid dysfunction fluctuations or local infections were observed in the 125I+TSH group. Overall, 125I+TSH combination therapy demonstrated a favorable safety profile with fewer neurological and metabolic complications compared with conventional treatment.

**Table 14:** Comparison of adverse events between groups.

Adverse events	125I+TSH group (n=110)	Control group (n=110)	*χ²*	*P*
None	93 (84.55%)	85 (77.27%)	1.883	0.170
Adverse events	17 (15.45%)	25 (22.73%)		
Pain ocal	13	7		
Hematoma minor	3	1		
RLN paresis	1	9		
Hypocalcemia	0	6		
Thyroid function shift	0	1		
Infection local	0	1		

Data are presented as n (%). Statistical significance was determined using χ² or Fisher’s exact test where appropriate.

## 4 Discussion

This study demonstrated that 125I seed implantation combined with TSH suppression significantly improved treatment outcomes and local control in postoperative recurrent or radioiodine-refractory DTC patients with cervical LNM. Compared with conventional therapy, the combined group exhibited approximately 50 percentage-point higher PR rates at 6 and 12 months, markedly accelerated Tg decline and lesion shrinkage, and achieved superior LRRFS and PFS, indicating a dual synergistic effect.

### 4.1 Comparison with previous studies

Previous studies reported local control rates of 60–80% for 125I seed implantation in locally recurrent thyroid cancer, but most were limited by small sample sizes and lacked evaluation of safety and dosimetric outcomes (14, 15). Our study, using a large sample with PSM analysis, confirmed the superior efficacy and safety of 125I implantation combined with TSH suppression (14). TSH suppression is a key component of postoperative management for DTC and acts by reducing circulating TSH levels, thereby inhibiting TSH-mediated stimulation of residual thyroid cancer cells. The combination of endocrine suppression with local radiotherapy may enhance radiosensitivity and limit tumor proliferation, contributing to improved biochemical remission and more durable lesion control. Our results (12-month PR ~74%, LRRFS 91.8%) outperform prior reports, likely due to multimodal optimization including image guidance, intraoperative dose planning, and post-operative dosimetric verification, ensuring precise seed distribution and uniform dosing. Additionally, TSH suppression reduces pro-proliferative signaling in thyroid cells, enhancing radiosensitivity and prolonging tumor control (16–18).

In addition to ¹²^5^I brachytherapy, several minimally invasive local therapies have been explored for the management of recurrent cervical lymph node metastases in DTC. Radiofrequency ablation (RFA) has shown promising results in selected cases; however, its efficacy may be limited for lesions located near critical structures such as the recurrent laryngeal nerve or major vessels. Ethanol injection therapy has also been reported as a potential option for small metastatic lymph nodes but often requires multiple sessions and may show variable long-term control (19). External beam radiotherapy provides broader regional coverage but is associated with greater radiation exposure to surrounding tissues (20). Compared with these approaches, ¹²^5^I seed implantation offers continuous low-dose irradiation with steep dose fall-off, enabling precise local control while minimizing damage to adjacent organs.

### 4.2 Potential mechanisms

The superior therapeutic efficacy observed in the ¹²^5^I+TSH group may be explained by several biological mechanisms. First, 125I seeds continuously deliver low-dose-rate γ radiation, inducing DNA double-strand breaks and apoptosis in tumor cells (21–23), thereby limiting local regenerative potential. Second, TSH suppression decreases tumor cell dependence on thyroid hormones, reducing metabolic activity and proliferation rate, which synergistically delays lesion progression at the molecular level (24). Furthermore, our dosimetric analysis demonstrated that D90 was positively correlated with V100 and planned dose and negatively correlated with seed displacement, highlighting the importance of adequate target coverage and precise seed placement for achieving optimal radiobiological effects, consistent with Leyrat et al. (25, 26). Bland–Altman analysis further confirmed high agreement between planned and measured doses (mean bias 0.29 Gy), supporting excellent reproducibility and dosimetric precision of image-guided 125I implantation.

### 4.3 Clinical implications

For RAI-refractory or surgically high-risk DTC patients, 125I implantation combined with TSH suppression offers a safe, minimally invasive, and effective alternative. This strategy provides targeted local tumor control while avoiding complications commonly associated with repeat cervical surgery, such as recurrent laryngeal nerve injury and hypocalcemia. In the present study, treatment-related adverse events were generally mild and transient, most commonly consisting of local pain or small hematomas at the implantation site. These findings indicate that the combined therapeutic strategy is well tolerated and may be particularly valuable for patients with surgically challenging lesions or high operative risk.

### 4.4 Limitations and future perspectives

Several limitations of this study should be acknowledged. First, although propensity score matching and multiple statistical models were used to enhance the robustness of the analysis, this study remains a retrospective single-center analysis, which may introduce residual confounding and selection bias. Although PSM improves comparability between groups, unmeasured variables and institutional treatment preferences may still influence outcomes. Second, the median follow-up duration was relatively short (approximately 12 months), which limits the ability to fully evaluate long-term survival outcomes and recurrence patterns. Additionally, molecular subtyping, tumor microenvironment, and radiobiological parameters were not included. Future multicenter prospective RCTs integrating radiomics and multi-omics are warranted to optimize dosimetric parameters and TSH targets, enabling personalized treatment strategies. In addition, radioiodine status was determined retrospectively based on available clinical and imaging records, which may introduce some classification uncertainty. Another limitation of this study is the absence of patient-reported outcomes. Increasing evidence suggests that patient-reported outcomes provide important insights into treatment-related symptom burden and quality of life, complementing traditional clinical endpoints in oncology trials (27). As highlighted by Bellino et al., incorporating patient-reported outcomes into clinical research can improve patient-centered evaluation and inform clinical decision-making. Patient-reported outcomes were not systematically collected in this retrospective cohort. Increasing evidence suggests that PROs provide valuable insights into treatment-related symptoms and quality of life and complement traditional clinical endpoints in oncology research. Future prospective studies should integrate validated quality-of-life instruments to better capture the patient experience associated with ¹²^5^I seed implantation therapy.

## 5 Conclusion

125I seed implantation combined with TSH suppression significantly improves local tumor control, accelerates biochemical remission, and prolongs recurrence-free survival in DTC patients with cervical LNM. The treatment demonstrates a favorable safety profile and represents a promising therapeutic option for patients with radioiodine-refractory disease or those who are unsuitable for repeat surgery. These findings provide new evidence supporting the integration of image-guided brachytherapy into the multidisciplinary management of recurrent thyroid cancer.

## Data Availability

The original contributions presented in the study are included in the article/**Supplementary Material**. Further inquiries can be directed to the corresponding author.
