# Supplementary material for: Efficacy and safety of 125I seed implantation combined with thyroid-stimulating hormone suppression therapy in cervical lymph node metastases of differentiated thyroid carcinoma
Source: Front Endocrinol (Lausanne). 2026 May 8;17:1747769. doi: 10.3389/fendo.2026.1747769 (PMC13193980; doi:10.3389/fendo.2026.1747769)
Supplement: Supplementary file 1 [file Table1.docx]

Supplementary Table S1 Treatment distribution in the control group

| Treatment | n | % |
| --- | --- | --- |
| Repeat surgery | 46 | 38.3% |
| Additional 131I therapy | 74 | 61.7% |
